# A Case of Fatty Tumor

**Published:** 1869-02

**Authors:** Generous L. Henderson

**Affiliations:** Kokomo, Indiana


ARTICLE IX.

### A CASE OF FATTY TUMOR.

By GENEROUS L. HENDERSON, M.D., Kokomo, Indiana.

Mrs. Rayles, æt. 65; small in stature, of a nervous temperament.

About 26 years ago, she first noticed a small tumor, the size of a hazelnut, under the right arm, below the axilla, and a little to the anterior.

It gradually grew in size, but without any serious pain, until within the past year, when it began to enlarge very rapidly, and ulcerated, with sharp, shooting pains.

It was now that it began to undermine her health, and that she was willing to undergo a surgical operation.

Gave her *tonics and support* freely, for a fortnight before the operation; also an anodyne occasionally, to allay the pain and to procure sleep.

On the 29th of October, we (Cole and Henderson) called, and found the tumor of an oval shape, somewhat flattened, and tolerably well defined; it had a soft, doughy feel.

Being assisted by Dr. Shoultz, we determined to operate. First chloroforming her in a supine position, we operated by rapidly making an *elliptical* incision, and carefully dissecting out all of the tumor, which was readily done, without much hemorrhage: the wound was brought together with *pins*, adhesive plaster, etc. (in fact, we do not use any other *suture*, because it does not absorb moisture, nor produce the irritation that a thread would; and, besides, it holds the parts more firmly in coaptation).

The extirpated tumor is seven (7) by eight (8) inches in diameter, and weighs 2 lbs. 2½ oz.

The wound did not have a tendency to very rapidly heal; but, by gently stimulating it a little with *carbolic acid*, and giving a good and generous diet, with tonics, it kindly healed, with but little suppuration.

She is now well and about her household duties, and enjoying a better state of health than she has for years before the operation.

